# New Insights on Bone Tissue and Structural Muscle-Bone Unit in Constitutional Thinness

**DOI:** 10.3389/fphys.2022.921351

**Published:** 2022-07-08

**Authors:** Mélina Bailly, Audrey Boscaro, Thierry Thomas, Léonard Féasson, Frédéric Costes, Bruno Pereira, Jorg Hager, Bruno Estour, Bogdan Galusca, Lore Metz, Daniel Courteix, David Thivel, Julien Verney, Natacha Germain

**Affiliations:** ^1^ Université Clermont Auvergne, CRNH, AME2P, Clermont-Ferrand, France; ^2^ Department of Rheumatology, Hôpital Nord, CHU, Saint-Étienne, France; ^3^ INSERM U1059, University of Lyon-Jean Monnet University, Saint-Étienne, France; ^4^ Inter-University Laboratory of Human Movement Biology (LIBM) EA 7424, Jean Monnet University, Saint-Étienne, France; ^5^ Department of Sport Medicine and Functional Explorations, CHU, Clermont-Ferrand, France; ^6^ Biostatistics Unit, Délégation à la Recherche Clinique et à l’Innovation (DRCI), Clermont-Ferrand, France; ^7^ Metabolic Health Nestlé Research, Lausanne, Switzerland; ^8^ Eating Disorders Addictions and Extreme Bodyweight Research Group (TAPE) EA 7423, Jean Monnet University, Saint-Étienne, France; ^9^ Division of Endocrinology, Diabetes Metabolism and Eating Disorders, CHU, Saint-Étienne, France

**Keywords:** bone microarchitecture, constitutional thinness, mechanical interaction, muscle-bone unit, skeletal muscle, weight gain resistance

## Abstract

While few studies pointed out low bone mineral densities in constitutionally thin women, little is known about potential explanations. The objective was to further explore bone architecture in both women and men with constitutional thinness to investigate their mechanical muscle-bone coupling (or uncoupling). Thirty constitutionally thin people and 31 normal weight controls participated in the study. Body composition, hip structural analysis, and trabecular bone score were assessed by dual-energy X-ray absorptiometry, bone architecture using high-resolution peripheral quantitative computed tomography, and muscle explorations through histological staining on muscle biopsies. Thirty-two out of the 48 indexes relative to density, geometry, texture, and architecture of bones were found significantly lower (*p* < 0.05) in constitutionally thin individuals compared with controls. This observation was particularly pronounced in constitutionally thin men. Bone microarchitecture was more altered in weight-supporting bone (tibia) than in non-weight-supporting (radius) bone, which might refer to a normal physiological adaptation (Frost’s mechanostat theory). Yet, the heat-maps of correlations analyses showed many alterations of body weight or muscle associations with bone parameters in constitutionally thin individuals contrary to controls. Present results might support the idea of intrinsic disturbances of bone cells independently to the small muscle structure, particularly in men.

## Graphical Abstract

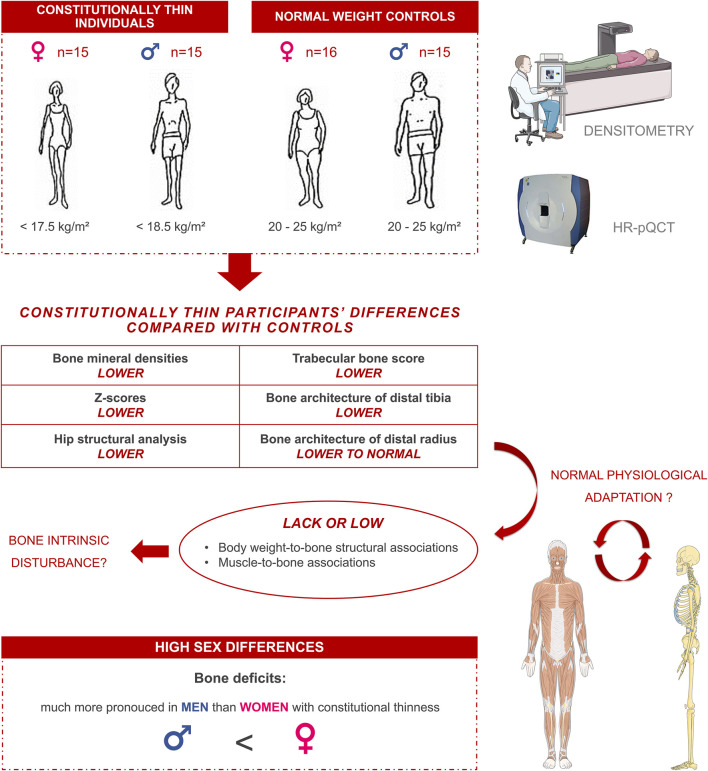

## 1 Introduction

Constitutional thinness (CT) is defined as a non-pathological state of underweight in the absence of apparent energy balance impairments (Bosy-Westphal et al., 2004; Germain et al., 2007; Galusca et al., 2018). People with CT are free of eating disorders, over-exercising behaviors or chronic diseases (Tagami et al., 2004; Bossu et al., 2007; Marra et al., 2009, 2019; Germain et al., 2014; Ling et al., 2019; Bailly et al., 2020c). Although CT seems to be characterized by normal energy intake and expenditure, individuals with CT remain at very low body mass index (BMI) below 18.5 kg/m^2^ and can reach very low values (such as 14 or 15 kg/m^2^) without any obvious physiological explanation (Bossu et al., 2007; Germain et al., 2007, 2009; Galusca et al., 2008, 2012, 2015). This atypical condition remains so underexplored that even its prevalence remains unclear. Yet, based on the data of Orthofer study, it may be calculated at 1.9% (Orthofer et al., 2020). To date, this uncommon phenotype remains poorly investigated with only about 40 clinical trials from 1950 (Bailly et al., 2020c, 2021). Based on such a limited number of studies, the absence of health concerns is not, however, robustly demonstrated, particularly according to studies assessing bone tissue (Galusca et al., 2008, 2009). In 2008, Galusca et al. (2008) showed that women with CT presented a low bone mineral density (BMD) of femoral neck and lumbar spine as low as patients with anorexia nervosa (AN). They reported that CT women displayed a lower bone resistance, a lower bone breaking strength, a lower bone cross-sectional area (CSA), and a markedly impaired bone microarchitecture in the distal tibia, compared with normal weight controls (NW) (Galusca et al., 2008). Up to 44% of CT women presented Z-scores below −2.0 (Galusca et al., 2008, 2009) which is considered as “below the expected range for age” according to the International Society for Clinical Densitometry (ISCD) (ISCD, 2019). However, the assessments of bone formation and resorption markers did not provide evidence of bone remodeling impairments in CT, contrary to AN (Galusca et al., 2008, 2009). A recent systematic review from our group (Bailly et al., 2021) showed that only six studies (Bosy-Westphal et al., 2004; Galusca et al., 2008, 2018; Fernández-García et al., 2009; Hasegawa et al., 2011; Estour et al., 2017) performed bone mineral content (BMC) and/or BMD assessments in CT and exclusively in women. All the studies which compared BMC or BMD between CT and AN found similar values between these populations (Galusca et al., 2008; Fernández-García et al., 2009; Estour et al., 2017). Only the study from Galusca et al. (2008) further explored architecture indexes in women with CT using high-resolution peripheral quantitative computed tomography (HR-pQCT).

While a lower BMD among people with CT seems today clearly admitted, its origin remains however uncertain. We suggest that this low BMD might either result from intrinsic bone disturbances or from normal physiological adaptations. Indeed, Harold Frost’s mechanostat theory stated that bone tissue is constantly adapting to biomechanical stimuli (Frost, 1987), and both low body weight and low muscle mass (MM) might lead to low biomechanical stimuli in CT (Bettis et al., 2018). Yet, it is now well-documented that environments with micro or hypo gravity such as those observed in space flights (Sibonga et al., 2007) or bed rest models (Kramer et al., 2017) result in an important decrease of bearing bones BMD (Williams et al., 2009; Ruggiu and Cancedda, 2015). On the opposite, hyper gravity models (Tominari et al., 2019) or elevated body weight (Albala et al., 1996) lead to an increase of BMD of weight-bearing bones. In addition, people with CT present low MM (Bossu et al., 2007; Marra et al., 2009, 2019; Galusca et al., 2018; Bailly et al., 2021) and low muscle fiber sizes (Galusca et al., 2018; Bailly et al., 2020b). We dedicated a previous paper to the analysis of muscle biopsies in CT showing a strong muscle fibers hypotrophy (Bailly et al., 2020b). Yet, bone and muscle tissues are indeed known to operate together synergistically as a system of pulley and levers through mechanical interactions, in which muscles supply load and bones provide attachments sites (Brotto and Bonewald, 2015; Goodman et al., 2015). Given this known relationship between bone and muscle tissues (Schoenau, 2005; Fricke and Schoenau, 2007), the low BMD in CT might, at least in part, results from a low muscle mechanical strength. Assuming that body weight (Ruggiu and Cancedda, 2015) as well as muscle contractions (Hamrick, 2010) are the main source of bone mechanical load, it might be of particular interest to interpret bone macro and microarchitecture in CT under consideration of both muscle structure and body weight.

The novelty of the present study was to explore muscle-bone unit at the structural and not at the functional level, in a population of adults with CT. The main objective of the present article was therefore to describe bone macro architecture, microarchitecture, geometry and texture parameters, in order to examine the physiological coupling of muscle and weight with bone tissue in CT vs. NW people, but also between the sexes.

## 2 Materials and Methods

### 2.1 Ethics

Investigations were conducted in accordance with the 1964 Helsinki declaration and registered in ClinicalTrials.gov with the following number: NCT02004821. The local research and ethics committee of Saint-Étienne, France (ANSM, 2013-A00590-45) have given their approval and all the participants have given a written informed consent. The clinical investigation was developed in partnership with Nestlé Institute of Health Science (NIHS), Switzerland.

### 2.2 Participants

From a total of 67 initially recruited participants, 61 young Caucasian subjects (18–35 years) finally performed the baseline measurements (Ling et al., 2016) [summary flowchart previously published (Ling et al., 2019, 2020)]. These healthy participants were assigned as follows: 15 women with CT (BMI < 17.5 kg/m^2^, 16.5 ± 0.8 kg/m^2^), 15 men with CT (BMI < 18.5 kg/m^2^; 17.4 ± 0.8 kg/m^2^), 16 NW women (BMI: 20–25 kg/m^2^; 23.0 ± 1.1 kg/m^2^), and 15 NW men (BMI: 20–25 kg/m^2^; 23.0 ± 1.2 kg/m^2^). CT subjects were recruited among outpatients consulting for bodyweight gain desire and were free of eating disorders (DSM-IV), as confirmed by psychiatric evaluations and Dutch Eating Behavior Questionnaire (DEBQ), Eating Disorder Examination (EDE) questionnaire, Eating Disorder Inventory (EDI), and Body Shape Questionnaire (BSQ). Their non-undernourishment was moreover supported by the normality of nutritional biomarkers such as free triiodothyronine (FT3) (5.93 pmol/L ± 1.97 in CT and 5.44 pmol/L ± 0.64 in NW participants; *p* = 0.52, *η* ^2^: 0.01) and insulin-like growth factor 1 (IGF-1) (238.0 µg/L ± 87.4 in CT and 253.0 µg/L ± 52.1 in NW participants; *p* = 0.37, *η* ^2^: 0.01). They had a low but stable bodyweight as confirmed by their personal weight history and women did not present any amenorrhea. None of the CT or NW participants presented any signs of chronic pathologies, congenital diseases, or severe progressive disorders. None of the participants neither reported intensive physical activity (more than three sessions of physical activity per week) nor presented over-exercising behavior according to the MONICA Optional Study of Physical Activity (MOSPA) questionnaire (Iqbal et al., 2006). They had no significant tobacco or alcohol consumptions (more than ten cigarettes per day or ten glasses of wine per week). For more information, we refer to the complete and detailed design of the protocol which has previously been published (Ling et al., 2016). In addition, we refer to a previous publication on the same cohort for the results specific to analyses of muscle biopsies (Bailly et al., 2020b).

### 2.3 Anthropometry, Body Composition, and Physical Activity

Standing height was measured to the nearest millimeter using a wall-mounted stadiometer and body mass was measured to the nearest 0.1 kg with a digital scale (ProDo, PD200M, Detecto, Webb City, MO, United States). Dual-energy X-ray absorptiometry (DXA) (LUNAR, DPX-L) was used to assess body composition—fat mass (FM), lean mass and BMC. Physical activity level (PAL) of the participants was recorded with an accelerometer (ActiHeart, CamNtech, Cambridge, United Kingdom) in free-living conditions over a 5-day period.

### 2.4 Bone Evaluation

#### 2.4.1 Bone mineral Density and Z-Scores

BMD measurements were performed using DXA with the same device at the lumbar spine (L1-L4), upper extremity of femur (including the femoral neck), Ward’s triangle, the intertrochanteric and femoral shaft regions. For BMD reporting in women prior to menopause and in men younger than age 50, the ISCD recommended the use of Z-scores rather than T-scores (ISCD, 2019). Z-scores below −2.0 are categorized as “below the expected range for age” and Z-scores above 2.0 are considered “within the expected range for age” (ISCD, 2019).

#### 2.4.2 Hip Structural Analysis and Trabecular Bone Score

DXA scans at femoral neck, intertrochanteric and femoral shaft regions were analyzed to assess hip bone geometry. Advanced hip structural analysis (HSA) technique, a validated method to assess hip bone geometry (Ackerman et al., 2013), used the properties of DXA images to derive hip geometric parameters. Hip axis length (HAL) represented the length of the femoral neck and head. Cross-sectional moment of inertia (CSMI) reflected the distribution of density around the femoral neck and reflected periosteal apposition which is related to bone resistance. CSA represented an index of resistance to axial forces taken following the axis from the center of mass to the superior neck margin along the narrow neck region. The neck shaft angle quantified the inclination between the femoral neck and the femoral shaft. The trabecular bone score (TBS) corresponded to a grey-level texture measurement which was directly applied to raw DXA images. This calculated score has been developed to be representative of bone quality and microarchitecture.

#### 2.4.3 Bone Architecture Investigation by HR-pQCT

Non-invasive HR-pQCT (XtremCT, Scanco Medical AG Basserdorfs, Switzerland) was used to explore specific parameters of bone architecture of the distal tibia and the distal radius of non-dominant limbs of participants (left for all participants except 2 of them). The subjects’ limbs were properly positioned and immobilized in the scanner. The techniques used for HR-pQCT have been previously detailed (Laib et al., 1998; Boutroy et al., 2005; Bouxsein et al., 2010; Schipilow et al., 2013; Whittier et al., 2020). Briefly, scans provided high-resolution images of a 9.02 mm section of the distal tibia and radius which allowed direct and derived measurements. A 2D detector array in combination with a 0.08 mm point-focus X-ray tube was used and enabled simultaneous acquisition of a stack of parallel computed tomography slices with a nominal resolution (voxel size) of 82 µm. The imaging principle was based on the interaction of X-rays with matter and the attenuation of radiation was related to the density of the matter, such as bones. X-ray attenuation data was acquired at multiple projections around the specimen allowing the reconstruction of 3D images (Whittier et al., 2020). With an effective radiation dose of only 3–5 µSv, this technique was considered as a low radiation dose procedure. HR-pQCT was used to assess BMD and bone microarchitecture parameters. Total, cortical, and trabecular areas respectively referred to the measurement of total CSA within the periosteal surface, total CSA within the cortical bone compartment, and total CSA within the trabecular compartment. These CSA measurements were calculated as the mean of CSA respective compartments in all image slices (Whittier et al., 2020). Average bone density (D100) and compact bone density (Dcomp) respectively refered to the average mineral density within the periosteal surface and to the average mineral density within the cortical compartment both calculated directly from the greyscale image data (Whittier et al., 2020). Cortical bone parameters were assessed through cortical thickness which was indirectly measured calculating the volume of the cortical compartment divided by the periosteal surface, and cortical perimeter which refered to the length of periosteal perimeter calculated as the mean of periosteal perimeter in all image slices. Trabecular bone density (Dtrab), meta trabecular bone density (Dmeta), and inner trabecular bone density (Dinn) respectively referred to average mineral density directly calculated from the greyscale image data of the total trabecular compartment, 40% of the trabecular compartment, and 60% of the trabecular compartment. Trabecular bone microarchitecture was assessed through different parameters (Whittier et al., 2020). Trabecular bone volume fraction was a derived measurement method referring to the ratio of segmented bone volume to total volume of trabecular compartment (tBV/TV), reported as percentages. The number of trabeculae (tTb.N) represented the average number of trabecular per unit length calculated as the mean inverse of the spacing between mid-axis (ridges) of the trabeculae. Trabecular thickness (tTb.Th) refered to the average thickness of trabeculae calculated as the ratio of tBV/TV to tTb.N. Trabecular separation (tTb.Sp) was the average distance between trabeculae calculating as following: (1 – tVB/TV)/tTb.N (derived measurement method). Inhomogeneity of trabecular network (tTb/N.SD) measured the standard deviation (SD) of the spacing between mid-axis (ridges) of the trabeculae, calculated as the SD of 1/tTb.N.

### 2.5 Muscle Evaluation

#### 2.5.1 Muscle Biopsies

Muscle biopsy samples of the right vastus lateralis were taken from the superficial portion of the muscle using a Weil-Blakesley forceps (Lawton, Tuttlingen, Germany) and a percutaneous technique (Henriksson, 1979; Féasson et al., 2002). Asepsis was performed, after shaving, with alcohol and iso-betadine followed by local anesthesia of the cutaneous and subcutaneous tissues (2% lidocaine, AstraZeneca, Rueil-Malmaison, France) and then by a small incision (<8 mm) to collect the muscle tissue. Five minutes of compression allowed to ensure hemostasis and the access was closed using sterile strips. Part of the biopsy with well-identified fascicles was oriented under a stereo microscope, mounted in an embedding medium (Cryomount, Histolab, Göteborg, Sweden), frozen in isopentane cooled to its freezing point in liquid nitrogen and stored in a freezer at −80°C until immunohistochemical analyses. Transverse sections 10-µm thick were cut at −18°C using a cryostat (CM 1950, Leica Biosystems, Wetzlar, Germany).

#### 2.5.2 Immunofluorescence

**FIGURE 1:**
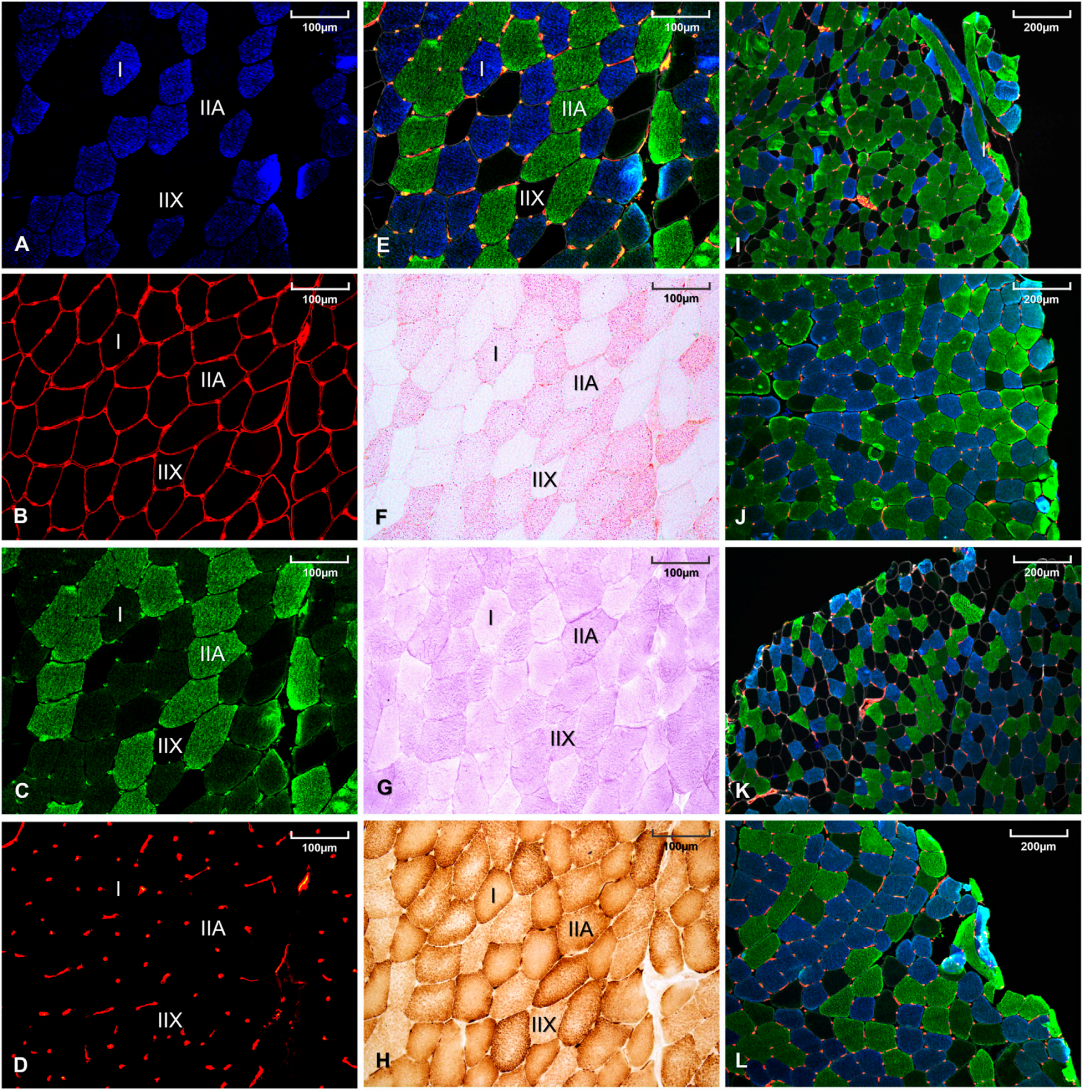
Histological staining of a same muscle sample using BA-F8 primary antibody **(A)**, 2E8 primary antibody **(B)**, BF-35 primary antibody **(C)**, anti-CD31-JC70A primary antibody **(D)**, merge of **(A–D)** images **(E)**, oil red O staining **(F)**, periodic acid Schiff staining **(G)**, diaminobenzidine staining combined with cytochrome-c oxidase activity **(H)**. Representative muscle sample from a women with constitutional thinness **(I)**, a normal weight women **(J)**, a men with constitutional thinness **(K)** and a normal weight men **(L)**.

Muscle fiber type, basal lamina, and capillaries identifications were performed by immunofluorescence on a single muscle transverse section. As detailed in Supplementary Data S1, monoclonal antibodies against myosin heavy chain (MHC) I (BA-F8) and all MHC but IIX (BF-35) were respectively conjugated to Alexa Fluor secondary antibodies 350 and 488 to simultaneously identify type I, IIA, and IIX muscle fibers. Anti-laminin 2E8 was conjugated to Alexa Fluor 633 to identify extracellular matrix allowing accurate assessment of fibers perimeter and Anti-CD31 was conjugated to Alexa Fluor 546 for capillaries identification (Supplementary Data S1). Immunohistological microscopic observations are displayed in Figure 1. This immunohistochemical staining technique was performed in two-steps to avoid cross-reactivity between BF-35 and anti-CD31 antibodies. This staining method has been previously described and validated (Bailly et al., 2020a). A slide scanner (Axio Scann.Z1, Carl Zeiss, Munich, Germany) was used for acquisition and ImageJ software (NIH, Bethesda, MD, United States) for image processing and analyses, as previously described (Bailly et al., 2020b). Muscle fibers CSA were only measured on the three main fiber types (I, IIA, IIX) and not on hybrid I-IIA, or IIA-IIX fibers. CSA measurements were performed on 150 fibers for each fiber type, except in the rare cases where the number of analyzable fibers was not sufficient. In order to have a representative index of the global muscle fiber size of a subject, an overall muscle fiber CSA was calculated as the sum of the products between percentages and mean areas of each fiber type. To indirectly refer to a global fiber size, the number of muscle fibers contained per muscle area was also determined without consideration of the fiber types. Capillary supply in skeletal muscle of participants was assessed using capillary density (number of capillaries per area), capillary-to-fiber ratio (ratio between the number of capillaries and the number of fibers per area), and the capillary contact index (number of capillaries in direct contact per independent fiber) (Harris, 2005) (Figure 1E).

#### 2.5.3 Energy Storage and Enzyme Activities

Intramuscular triglycerides (IMTG) were revealed by oil red O staining and muscle glycogen by periodic acid Schiff staining [previously detailed (Bailly et al., 2020b), Figures 1F,G]. Histological cytochrome-c oxidase (COx) activity (Figure 1H) was identified by coupling the enzyme reaction with 3,3-diaminobenzidine staining. Optical densities were obtained after images conversion to grayscale and quantification of the mean gray intensity per muscle fiber using ImageJ Software. Global storage indexes were obtained by multiplying the percentages of area occupied by each fiber type by their respective mean optical densities (arbitrary unit). Optical density was independent of the fiber CSA since the value represented the average grey density of each individual pixel contained within the fiber delineation. Enzyme activities were measured as the maximal slope of enzyme reactions with substrates in excess at 37°C using spectrophotometry with a microplate reader (Clariostar, BMG Labtech, Ortenberg, Germany).

### 2.6 Statistics

Continuous data are presented as means ± SD. The assumption of normality was systematically verified using Shapiro-Wilk test. Multiple linear models were performed to assess the main effects (Group, Sex, interaction Group × Sex) on general characteristics of the population, bone texture, and bone architecture parameters (Tables 1–3), taking into account prespecified covariates (age and PAL). Subgroups analyses according to sex were conducted with same statistical approaches, i.e., linear model taking into account age and PAL covariates. The normality of residuals from these models were analyzed as aforementioned. In the absence of normal distribution, logarithmic transformation of dependent variables were used to achieve normality. For the comparisons concerning age and height variables, which did not require covariates adjustments, Student t-test or non-parametric Mann-Whitney test were performed according to assumptions of *t*-test. The associations between continuous variables (such as bone parameters, anthropometry, and skeletal muscle parameters) were estimated using Pearson or Spearman correlation coefficients (depending on the statistical distribution).

**TABLE 1:** Anthropometry, body composition, densitometry, bone geometry and bone texture in women and men with constitutional thinness compared with normal weight controls.

	Both sexes		Women		Men		Group	Sex	Interaction group × sex
	CT (*n* = 31)	NW (*n* = 30)	CT (*n* = 15)	NW (*n* = 16)	CT (*n* = 15)	NW (*n* = 15)	*p*-value(η^2^)	*p*-value(η^2^)	*p*-value(η^2^)
General characteristics									
Age (yr)	25.5 ± 4.5	23.1 ± 2.9	27.4 ± 4.6^c^	22.4 ±2.8	23.6 ± 3.8	23.9 ± 2.9	0.014 (0.10)	0.20 (0.03)	0.006 (0.13)
Weight (kg)	48.3 ± 6.7	68.6 ± 8.5	42.8 ± 4.5^c^	62.9 ± 4.8	53.8 ± 2.9^c^	74.8 ± 7.3	<0.001 (0.76)	<0.001 (0.57)	0.85 (0.00)
Height (cm)	168.2 ± 9.3	172.6 ± 9.8	160.7 ± 6.4^a^	165.5 ± 6.4	175.8 ± 4.0^a^	180.1 ± 6.6	0.0042 (0.14)	<0.001 (0.62)	0.90 (0.00)
BMI (kg/m^2^)	16.97 ± 0.89	22.99 ± 1.14	16.53 ± 0.77^c^	22.96 ± 1.12	17.41 ± 0.80^c^	23.01 ± 1.20	<0.001 (0.89)	0.062 (0.06)	0.12 (0.04)
IGF-1 (µg/L)	238.0 ± 87.4	253.0 ± 52.1	239.2 ± 66.7^d^	274.2 ± 38.3	236.8 ± 106.6	231.9 ± 56.5	0.37 (0.01)	0.20 (0.03)	0.91 (0.00)
FT3 (pmol/L)	5.93 ± 1.97	5.44 ± 0.64	6.20 ± 2.75	5.45 ± 0.58	5.67 ± 0.58	5.43 ± 0.73	0.52 (0.01)	0.63 (0.00)	0.40 (0.01)
Total fat mass (%)	19.4 ± 4.8	26.4 ± 7.5	23.3 ± 2.9^c^	31.6 ± 4.0	15.5 ± 2.3^a^	20.9 ± 6.4	<0.001 (0.34)	<0.001 (0.57)	0.55 (0.01)
Total lean mass (kg)	39.1 ± 6.9	49.8 ± 9.4	32.8 ± 3.3^c^	42.1 ± 3.5	45.3 ± 2.2^c^	58.1 ± 6.0	<0.001 (0.60)	<0.001 (0.78)	0.29 (0.02)
Legs lean mass (kg)	11.23 ± 2.31	15.83 ± 3.06	9.21 ± 1.10^c^	13.40 ± 1.24	13.24 ± 1.04^c^	18.42 ± 2.12	<0.001 (0.68)	<0.001 (0.73)	0.47 (0.01)
Physical activity level (PAL)	1.57 ± 0.22	1.76 ± 0.22	1.60 ± 0.22	1.72 ± 0.18	1.55 ± 0.24^b^	1.81 ± 0.26	0.010 (0.11)	0.88 (0.00)	0.11 (0.05)
Total bone mineral content (kg)	2207 ± 308	2758 ± 434	2004 ± 261^a^	2427 ± 245	2410 ± 199^c^	3111 ± 283	<0.001 (0.48)	<0.001 (0.55)	0.074 (0.06)
Bone mineral density (BMD)									
Total body (g/cm^2^)	1.028 ± 0.076	1.169 ± 0.078	1.029 ± 0.078^a^	1.129 ± 0.070	1.026 ± 0.078^c^	1.211 ± 0.065	<0.001 (0.43)	0.074 (0.06)	0.049 (0.07)
Lumbar spine L1-L4 (g/cm^2^)	1.045 ± 0.108	1.178 ± 0.096	1.074 ± 0.111^a^	1.190 ± 0.111	1.016 ± 0.100^b^	1.164 ± 0.077	<0.001 (0.27)	0.20 (0.03)	0.94 (0.00)
Legs (g/cm^2^)	1.116 ± 0.103	1.299 ± 0.129	1.093 ± 0.098^d^	1.215 ± 0.100	1.139 ± 0.105^c^	1.388 ± 0.093	<0.001 (0.41)	<0.001 (0.23)	0.039 (0.08)
Total femur (neck, trochanter, ward, diaphysis) (g/cm^2^)	0.935 ± 0.118	1.092 ± 0.096	0.932 ± 0.135^d^	1.072 ± 0.108	0.937 ± 0.102^c^	1.112 ± 0.080	<0.001 (0.31)	0.39 (0.01)	0.55 (0.01)
Femoral neck (g/cm^2^)	0.944 ± 0.134	1.093 ± 0.098	0.969 ± 0.143	1.073 ± 0.095	0.920 ± 0.123^c^	1.113 ± 0.101	<0.001 (0.25)	0.94 (0.00)	0.20 (0.03)
Femoral trochanter (g/cm^2^)	0.704 ± 0.124	0.877 ± 0.100	0.676 ± 0.142^a^	0.840 ± 0.108	0.731 ± 0.099^c^	0.916 ± 0.076	<0.001 (0.36)	0.021 (0.09)	0.71 (0.00)
Z-scores									
Total body	0.7 ± 0.6	0.8 ± 0.6	0.7 ± 0.6	0.8 ± 0.6	NA	NA	NA	NA	NA
Lumbar spine L1-L4	−0.6 ± 0.9	−0.2 ± 0.8	−0.3 ± 0.9	0.0 ± 0.9	−0.9 ± 0.9	−0.4 ± 0.6	0.072 (0.07)	0.052 (0.08)	0.99 (0.00)
Total femur (neck, trochanter, ward, diaphysis)	−0.4 ± 1.0	0.3 ± 0.7	−0.2 ± 1.1	0.4 ± 0.9	−0.6 ± 0.9^a^	0.3 ± 0.5	0.004 (0.15)	0.50 (0.01)	0.69 (0.00)
Femoral neck	−0.2 ± 1.2	0.4 ± 0.7	0.2 ± 1.1	0.4 ± 0.8	-0.7 ± 1.0^a^	0.4 ± 0.7	0.014 (0.11)	0.11 (0.05)	0.24 (0.03)
Femoral trochanter	−0.9 ± 1.1	0.2 ± 0.8	−0.7 ± 1.3	0.3 ± 1.0	−1.0 ± 1.0^b^	0.1 ± 0.6	<0.001 (0.23)	0.36 (0.02)	0.92 (0.00)
HSA—Hip structural analysis									
HAL (mm)—Hip axis length	108.5 ± 10.4	114.7 ± 10.2	100.9 ± 5.6^a^	106.6 ± 6.1	116.6 ± 8.0^b^	123.4 ± 5.2	0.0012 (0.18)	<0.001 (0.63)	0.82 (0.00)
CSMI (cm^4^)—Cross-sectional moment of inertia	0.942 ± 0.258	1.287 ± 0.463	0.772 ± 0.202^d^	0.927 ± 0.153	1.124 ± 0.174^c^	1.670 ± 0.358	<0.001 (0.30)	<0.001 (0.59)	0.020 (0.10)
CSA (mm^2^)—Cross-sectional area	137.5 ± 18.4	170.9 ± 25.3	131.9 ± 19.8^a^	153.8 ± 13.7	143.6 ± 15.2^c^	189.1 ± 22.1	<0.001 (0.40)	<0.001 (0.32)	0.034 (0.08)
NSA (°)—Neck shaft angle theta	127.3 ± 4.2	128.1 ± 3.9	125.9 ± 3.9^d^	127.3 ± 3.6	128.8 ± 4.1	129.1 ± 4.0	0.11 (0.05)	0.021 (0.10)	0.95 (0.00)
TBS—Trabecular bone score									
TBS L1-L4—Trabecular bone score	1.34 ± 0.08	1.44 ± 0.07	1.35 ± 0.08^c^	1.47 ± 0.06	1.34 ± 0.08	1.41 ± 0.06	<0.001 (0.29)	0.089 (0.06)	0.048 (0.07)

a : *p* < 0.05 CT vs. NW within women or men.

b : *p* < 0.01 CT vs. NW within women or men.

c : *p* < 0.001 CT vs. NW within women or men.

d : Trend for 0.05 < *p* < 0.1 CT vs. NW within women or men.

The statistical analyses were performed using SPSS software (IBM Corp. Released 2011. Version 20.0. Armonk, NY: IBM Corp.) and the graphs were carried out with GraphPad Prism software (version 5.0 for Windows, La Jolla, CA United States). The tests were two-sided, with a type-I error set at 5%. A specific attention was paid on the magnitude of differences and to the clinical relevance. Eta squared effect sizes (*η* ^2^) were presented.

## 3 Results

### 3.1 Characteristics of Participants

As expected, participants with CT displayed a markedly lower BMI (−26%), total lean mass (−22%), and total bone mineral content (−20%) compared with NW people (*p* < 0.001). The same observation of a lower BMI, total lean mass, and total bone mineral content in CT vs. NW was also obtained when sexes were analyzed apart (Table 1). The total percentage of FM was found lower in CT women compared with NW women (23.3% ± 2.9 vs. 31.6% ± 4.0, *p* < 0.001) and lower in CT men compared with NW men (15.5% ± 2.3 vs. 20.9% ± 6.4, *p* = 0.014).

### 3.2 Muscle Atrophy and Oxidative Profile

All the results which specifically concerned the morphology, fiber typing, or biochemistry of the muscle tissue (independently of the muscle-bone unit) have already been published in a dedicated analysis (Bailly et al., 2020b). Histological analyses had shown low percentages of muscle area occupied by type I fibers (*p* = 0.044), high proportions of type IIX fibers (*p* = 0.033), a marked muscle hypotrophy (−20%, *p* < 0.001), and a low capillary supply (capillary-to-fiber ratio: −19%, *p* < 0.001) (Bailly et al., 2020b). Both IMTG and glycogen contents were found lower in CT compared with controls for type I fibers (respectively −17%, *p* = 0.002, −6%, *p* = 0.008) and type IIA fibers (respectively −14%, *p* = 0.048, −5%, *p* = 0.015). Participants with CT also presented a lower citrate synthase activity (−18%, *p* = 0.010). These findings suggest a low muscle energy storage and might be indicative of a low oxidative capacity in CT (Bailly et al., 2020b).

### 3.3 Bone Results

#### 3.3.1 Low Bone Mineral Densities

Total BMD was found to be lower in CT vs. NW participants (*p* < 0.001, *η* ^2^: 0.43). Both CT women and men presented lower total BMD values compared with NW women and men (1.029 g/cm^2^ ± 0.078 vs. 1.129 g/cm^2^ ± 0.070, *p* = 0.044 for women’ comparisons and 1.026 g/cm^2^ ± 0.078 vs. 1.211 g/cm^2^ ± 0.065, *p* < 0.001 for men’ comparisons). BMD from lumbar spine L1-L4, legs, total femur (neck, trochanter, ward, diaphysis), femoral neck, and femoral trochanter were all found lower in CT vs. NW with robust *p*-values (*p* < 0.001 for all these parameters). Men with CT presented a highly significant lower BMD compared with controls (lumbar spine: −13%, *p* = 0.0059, legs: −18%, *p* < 0.001, total femur: −16%, *p* < 0.001, femoral neck: −17%, *p* < 0.001, femoral trochanter: −20%, *p* < 0.001) whereas women with CT reached the significance only for some of these region sites (lumbar spine: −10%, *p* = 0.019, femoral trochanter: −19%, *p* = 0.023) (Table 1). Lower Z-scores were obtained in CT vs. NW participants for total femur, femoral neck and femoral trochanter. CT men presented Z-scores of −0.6 ± 0.9 for total femur, −0.7 ± 1.0 for femoral neck and −1.0 ± 1.0 for femoral trochanter which appeared significantly lower than NW men (respectively *p* = 0.010, *p* = 0.010, and *p* = 0.0013). Women with CT did not differ from NW women for either total or regionalized Z-scores (lumbar spine, total femur, femoral neck, and femoral trochanter).

#### 3.3.2 Impairments in Bone Geometry and Texture

The hip structural analysis revealed lower values in CT participants vs. NW participants for HAL, CSMI, and CSA whereas the neck shaft angle theta was not found different. HAL and CSA were found lower for both CT men (respectively *p* = 0.0090, *η* ^2^: 0.24; *p* < 0.001, *η* ^2^: 0.49) and CT women (respectively *p* = 0.022, *η* ^2^: 0.18, *p* = 0.037, *η* ^2^: 0.16) compared with their NW counterparts. Although CSMI was found 33% lower for CT men compared with NW men (1.124 cm^4^ ± 0.174 vs. 1.670 cm^4^ ± 0.358, *p* < 0.001), the same observation did not appear significant in CT men for L1-L4 TBS despite their low values. On the opposite, CT women presented a lower L1-L4 TBS compared with NW women (1.35 ± 0.08 vs. 1.47 ± 0.06, *p* < 0.001, *η* ^2^: 0.43), but the level of significance was not reached for CSMI comparison between CT and NW women (−17%, *p* = 0.064).

#### 3.3.3 A Bone Architecture Strongly Impacted in the Distal Tibia (HR-pQCT)

All dimensional parameters of distal tibia were found lower in CT vs. NW people: total area (−15%, *p* < 0.001), cortical area (−22%, *p* < 0.001), trabecular area (−13%, *p* = 0.022), cortical thickness (−13%, *p* < 0.001) and cortical perimeter (−10%, *p* = 0.034) (Table 2). All of these dimensional parameters were found lower in CT vs. NW men, with particularly large differences for total area (−15%, *p* < 0.001), cortical area (−27%, *p* < 0.001) and cortical thickness (−21%, *p* < 0.001). These differences were less robustly observed between CT women and NW women: −16% and *p* = 0.091 for total area, −16% and *p* = 0.028 for cortical area, and insignificant for cortical thickness. Compact bone density of the distal tibia was found similar between CT and NW participants without distinction between men and women as well as considering sex distinction (Table 2). Average and trabecular BMD of the distal tibia were found lower in CT men (−16%, *p* = 0.0067 and −20%, *p* = 0.0082) compared with NW men, whereas the low values obtained in CT women were not found significantly different from their NW counterpart. Inner trabecular BMD was found particularly lower in CT vs. NW participants (−26%, *p* = 0.0067 for CT males, −23%, *p* = 0.086 for CT females, −25%, *p* < 0.001 for both sexes). The meta-to-inner trabecular BMD ratio was observed higher in CT vs. NW people (+18%, *p* = 0.010 for CT men, +24%, *p* = 0.039 for CT women, +21%, *p* = 0.0021 for both sexes). Regarding trabecular microarchitecture, the number of trabeculae was found lower in both CT vs. NW men (1.59 mm^−1^ ± 0.27 vs. 2.04 mm^−1^ ± 0.22, *p* < 0.001) and CT vs. NW women (1.51 mm^−1^ ± 0.24 vs. 1.86 mm^−1^ ± 0.25, *p* = 0.0078). Higher trabecular separation was observed in both CT vs. NW men and CT vs. NW women (respectively +38%, *p* < 0.001; +29%, *p* = 0.017). Inhomogeneity of network was also higher in CT compared with NW people (+43%, *p* = 0.0038 for CT men; +30%, *p* = 0.022 for CT women). However, trabecular thickness was not found different between CT and NW participants, neither for women nor for men. Trabecular bone volume to tissue volume was found lower in CT vs. NW people. After separation by sex, this result was only significant for CT vs. NW men (*p* = 0.0084) and not for CT vs. NW women (Table 2).

**TABLE 2:** Bone architecture parameters of the distal tibia assessed using high resolution peripheral quantitative computed tomography in women and men with constitutional thinness compared with normal weight participants.

	Both sexes		Women		Men		Group	Sex	Interaction group × sexes
	CT (*n* = 31)	NW (*n* = 30)	CT (*n* = 15)	NW (*n* = 16)	CT (*n* = 15)	NW (*n* = 15)	*p*-value (η^2^)	*p*-value (η^2^)	*p*-value (η^2^)
Tibia—HR-pQCT									
**Dimensional parameters**									
Total area (mm^2^)	647 ± 121	758 ± 135	558 ± 91^d^	661 ± 99	736 ± 71^c^	861 ± 79	<0.001 (0.20)	<0.001 (0.54)	0.55 (0.01)
Cortical area (mm^2^)	107 ± 17	137 ± 29	102 ± 15^a^	121 ± 28	112 ± 18^c^	153 ± 19	<0.001 (0.41)	<0.001 (0.29)	0.42 (0.01)
Trabecular area (mm^2^)	537 ± 116	618 ± 125	454 ± 88	536 ± 106	620 ± 76^a^	706 ± 73	0.022 (0.09)	<0.001 (0.48)	0.69 (0.00)
Ct.Th (mm)—Cortical thickness	1.09 ± 0.18	1.26 ± 0.27	1.12 ± 0.17	1.19 ± 0.33	1.07 ± 0.19^c^	1.34 ± 0.14	<0.001 (0.23)	0.13 (0.04)	0.34 (0.02)
Ct.Pm (mm)—Cortical perimeter	98.4 ± 9.5	109.7 ± 19.0	91.4 ± 7.6	105.5 ± 25.5	105.4 ± 5.1^b^	114.2 ± 5.8	0.034 (0.08)	0.0071 (0.13)	0.64 (0.00)
**Bone mineral density (BMD)**									
D100 (mg HA/cm^3^)—Average	295 ± 49	332 ± 43	301 ± 50	324 ± 53	288 ± 49^b^	341 ± 26	0.0011 (0.18)	0.49 (0.01)	0.55 (0.01)
Dcomp (mg HA/cm^3^)—Compact	893 ± 45	889 ± 28	919 ± 30	899 ± 28	868 ± 44	879 ± 25	0.42 (0.01)	<0.001 (0.21)	0.89 (0.00)
Dtrab (mg HA/cm^3^)—Trabecular	167 ± 40	203 ± 31	156 ± 40	185 ± 21	177 ± 39^b^	222 ± 27	<0.001 (0.19)	<0.001 (0.19)	0.37 (0.02)
Dmeta (mg HA/cm^3^)—Meta trabecular	238 ± 45	272 ± 33	228 ± 45	253 ± 27	247 ± 43^a^	292 ± 26	0.0052 (0.14)	0.0030 (0.15)	0.27 (0.02)
Dinn (mg HA/cm^3^)—Inner trabecular	118 ± 39	157 ± 32	107 ± 38^d^	139 ± 22	130 ± 37^b^	176 ± 30	<0.001 (0.21)	<0.001 (0.20)	0.50 (0.01)
Meta/Inn—Ratio meta / inner trabecular	2.15 ± 0.48	1.77 ± 0.25	2.31 ± 0.55^a^	1.85 ± 0.26	1.99 ± 0.35^a^	1.69 ± 0.20	0.0021 (0.16)	0.0077 (0.12)	0.48 (0.01)
**Microarchitecture**									
tBV/TV (%)—Trabecular bone volume to tissue volume	13.9 ± 3.4	16.9 ± 2.6	13.0 ± 3.4	15.4 ±1.8	14.8 ± 3.3^b^	18.5 ± 2.3	<0.001 (0.18)	<0.001 (0.19)	0.36 (0.02)
tTb.N (1/mm)—Number of trabeculae	1.55 ± 0.26	1.95 ± 0.25	1.51 ± 0.24^b^	1.86 ± 0.25	1.59 ± 0.27^c^	2.04 ± 0.22	<0.001 (0.32)	0.064 (0.06)	0.67 (0.00)
tTb.Th (mm)—Trabecular thickness	0.090 ± 0.016	0.087 ± 0.012	0.087 ± 0.019	0.084 ± 0.014	0.093 ± 0.012	0.091 ± 0.009	0.56 (0.01)	0.035 (0.08)	0.95 (0.00)
tTb.Sp (mm)—Trabecular separation	0.576 ± 0.124	0.434 ± 0.064	0.595 ± 0.116^a^	0.462 ± 0.064	0.557 ± 0.132^c^	0.405 ± 0.052	<0.001 (0.30)	0.034 (0.08)	0.58 (0.01)
tTb.1/N.SD (mm)—Inhomogeneity of network (SD of 1/Tb.N)	0.269 ± 0.070	0.198 ± 0.043	0.274 ± 0.054^a^	0.211 ± 0.048	0.265 ± 0.085^b^	0.185 ± 0.034	<0.001 (0.20)	0.35 (0.02)	0.47 (0.01)

a : *p* < 0.05 CT vs. NW within women or men.

b : *p* < 0.01 CT vs. NW within women or men.

c : *p* < 0.001 CT vs. NW within women or men.

d : Trend for 0.05 < *p* < 0.1 CT vs. NW within women or men.

#### 3.3.4 A Bone Architecture Less Impacted in the Distal Radius (HR-pQCT)

Detailed results of the distal radius bone microarchitecture are displayed in Table 3. Without separation by sex, results showed significant lower values in CT vs. NW participants for total area of the distal radius, cortical area, trabecular area, and cortical thickness. All the other microarchitecture parameters of the distal radius were not found different between CT and NW people, except for 3 parameters which tended to be lower in CT (average BMD, meta trabecular BMD, and number of trabeculae). CT women did not present significant differences compared with NW women on any of the radius microarchitecture parameters. Despite not reaching significance, absolute BMD values in CT were systematically observed slightly below those of NW subjects (Table 3). Among men, many parameters were found lower in CT vs. NW men: total area of radius (−15%, *p* = 0.017), cortical area (−21%, *p* = 0.0043), cortical thickness (−17%, *p* = 0.034), trabecular BMD (−17%, *p* = 0.028), meta trabecular BMD (−12%, *p* = 0.026), inner trabecular BMD (−22%, *p* = 0.034), and trabecular bone volume to tissue volume (−17%, *p* = 0.028).

**TABLE 3:** Bone architecture parameters of the distal radius assessed using high resolution peripheral quantitative computed tomography in women and men with constitutional thinness compared with normal weight participants.

	Both sexes		Women		Men		Group	Sex	Interaction group × sex
	CT (*n* = 31)	NW (*n* = 30)	CT (*n* = 15)	NW (*n* = 16)	CT (*n* = 15)	NW (*n* = 15)	*p*-value (η^2^)	*p*-value (η^2^)	*p*-value (η^2^)
Radius—HR-pQCT									
**Dimensional parameters**									
Total area (mm^2^)	269 ± 52	307 ± 69	233 ± 41^c^	260 ± 43	305 ± 34^a^	358 ± 54	0.0023 (0.16)	<0.001 (0.53)	0.81 (0.00)
Cortical area (mm^2^)	51 ± 11	62 ± 13	47 ± 8^c^	55 ± 12	56 ± 11^b^	70 ± 9	<0.001 (0.26)	<0.001 (0.31)	0.43 (0.01)
Trabecular area (mm^2^)	212 ± 49	240 ± 62	182 ± 40	201 ± 45	242 ± 37^c^	281 ± 51	0.033 (0.08)	<0.001 (0.43)	0.91 (0.00)
Ct.Th (mm)—Cortical thickness	0.72 ± 0.17	0.84 ± 0.20	0.72 ± 0.17	0.79 ± 0.25	0.73 ± 0.18^a^	0.88 ± 0.11	0.012 (0.11)	0.21 (0.03)	0.57 (0.01)
Ct.Pm (mm)—Cortical perimeter	73.4 ± 19.5	77.4 ± 23.2	68.1 ± 17.8	74.6 ± 32.1	78.7 ± 20.2	80.3 ± 5.9	0.48 (0.01)	0.17 (0.03)	0.76 (0.00)
**Bone mineral density (BMD)**									
D100 (mg HA/cm^3^)—Average	315 ± 56	340 ± 55	321 ± 55	335 ± 70	308 ± 57	345 ± 34	0.065 (0.06)	0.99 (0.00)	0.32 (0.02)
Dcomp (mg HA/cm^3^)—Compact	855 ± 62	866 ± 45	879 ± 44	880 ± 48	830 ± 69	851 ± 37	0.11 (0.05)	0.016 (0.10)	0.91 (0.00)
Dtrab (mg HA/cm^3^)—Trabecular	164 ± 33	184 ± 32	159 ± 31	166 ± 30	169 ± 36^a^	203 ± 23	0.12 (0.04)	0.0076 (0.12)	0.045 (0.07)
Dmeta (mg HA/cm^3^)—Meta trabecular	227 ± 31	246 ± 28	225 ± 28	232 ± 26	229 ± 35^a^	260 ± 24	0.088 (0.05)	0.062 (0.06)	0.048 (0.07)
Dinn (mg HA/cm^3^)—Inner trabecular	121 ± 36	141 ± 36	114 ± 34	120 ± 33	127 ± 37^a^	164 ± 24	0.16 (0.04)	0.0018 (0.17)	0.049 (0.07)
Meta/Inn—Ratio meta / inner trabecular	2.00 ± 0.45	1.83 ± 0.40	2.11 ± 0.52	2.05 ± 0.45	1.89 ± 0.36^c^	1.60 ± 0.14	0.71 (0.00)	0.0031 (0.15)	0.16 (0.04)
**Microarchitecture**									
tBV/TV (%)—Trabecular bone volume to tissue volume	13.7 ± 2.8	15.3 ± 2.7	13.3 ± 2.6	13.8 ± 2.5	14.1 ± 3.0^a^	16.9 ± 1.9	0.12 (0.04)	0.0077 (0.12)	0.046 (0.07)
tTb.N (1/mm)—Number of trabeculae	1.71 ± 0.28	1.90 ± 0.24	1.66 ± 0.23	1.82 ± 0.21	1.76 ± 0.32	1.98 ± 0.24	0.089 (0.05)	0.099 (0.05)	0.50 (0.01)
tTb.Th (mm)—Trabecular thickness	0.080 ± 0.010	0.080 ± 0.010	0.080 ± 0.011	0.076 ± 0.009	0.080 ± 0.009	0.086 ± 0.008	0.88 (0.00)	0.033 (0.08)	0.022 (0.09)
tTb.Sp (mm)—Trabecular separation	0.521 ± 0.112	0.454 ± 0.070	0.534 ± 0.094	0.481 ± 0.072	0.508 ± 0.129^c^	0.426 ± 0.058	0.11 (0.05)	0.084 (0.05)	0.37 (0.02)
tTb.1/N.SD (mm)—Inhomogeneity of network (SD of 1/Tb.N)	0.230 ± 0.077	0.186 ± 0.041	0.243 ± 0.081	0.200 ± 0.045	0.216 ± 0.073	0.172 ± 0.030	0.12 (0.04)	0.078 (0.06)	0.53 (0.01)

a : *p* < 0.05 CT vs. NW within women or men.

b : *p* < 0.01 CT vs. NW within women or men.

c : Trend for 0.05 < *p* < 0.1 CT vs. NW within women or men.

### 3.4 Muscle-Bone Mechanical Unit

#### 3.4.1 Absence of Correlation Between Body Mass and Bone Mineral Density in Constitutional Thinness

**FIGURE 2:**
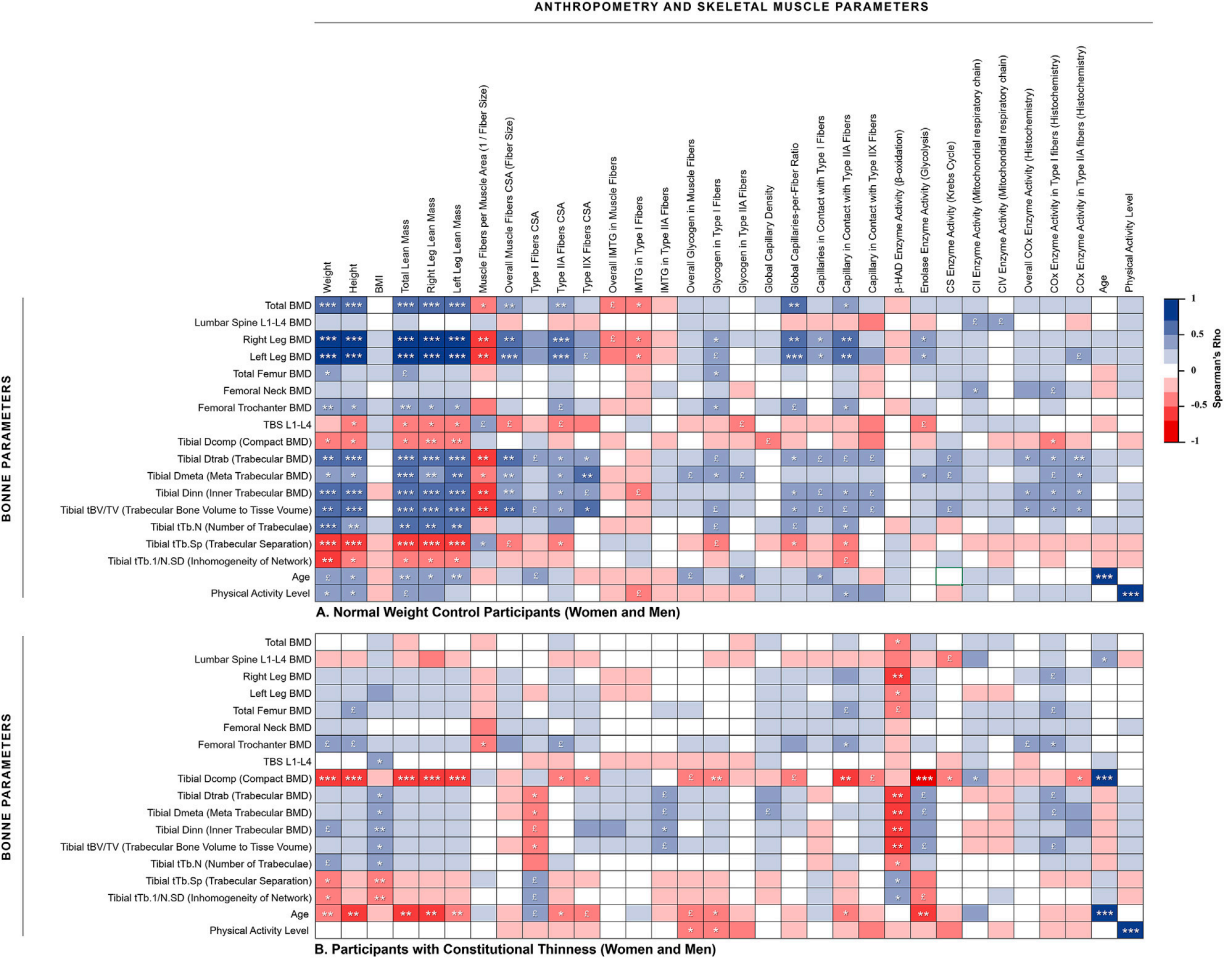
Heat map of correlations between muscle and bone assessments. **(A)** Normal weight control participants (women and men); **(B)** Participants with constitutional thinness (women and men). *p < 0.05, **p < 0.01, ***p < 0.001, £ 0.05 < *p* < 0.1 (trend). BMD, bone mineral density; BMI, body mass index; COx, cytochrome-c oxidase; CS, citrate synthase; CSA, cross-sectional area; IMTG, intramuscular triglycerides; TBS, trabecular bone score

The Figure 2 presents a heat map of the detailed associations between specific bone parameters (rows) and body mass / skeletal muscle parameters (columns) in order to further investigate the bone-to-muscle mechanical relationships. NW participants showed strong positive associations between their body weight and BMD from different sites whereas no such associations were observed for CT participants (Figure 2). For instance, body mass to total BMD relationship was observed positive and strong for NW people (rhô = 0.68, *p* < 0.001) contrary to CT participants (rhô = 0.02, *p* = 0.92). The same heat maps separating women from men results were respectively displayed in Supplementary Data S2, S3. The absence of weight to BMD associations in CT seems to be rather due to men results since negative weak associations were obtained in men with CT whereas moderate positive associations were observed in women with CT. For instance, body weight to total BMD relationship was observed moderate and positive for CT women (rhô = 0.53, *p* = 0.042) contrary to CT men (rhô = −0.11, *p* = 0.69). Moderate to strong positive associations between height and BMD were observed in NW participants (Figure 2) contrary to individuals with CT. Several height to tibia BMD associations even appeared negatively correlated in men with CT (Supplementary Data S3b).

#### 3.4.2 No Associations Between Muscle Mass / Fibers Size and Bone Parameters in Constitutional Thinness

**FIGURE 3:**
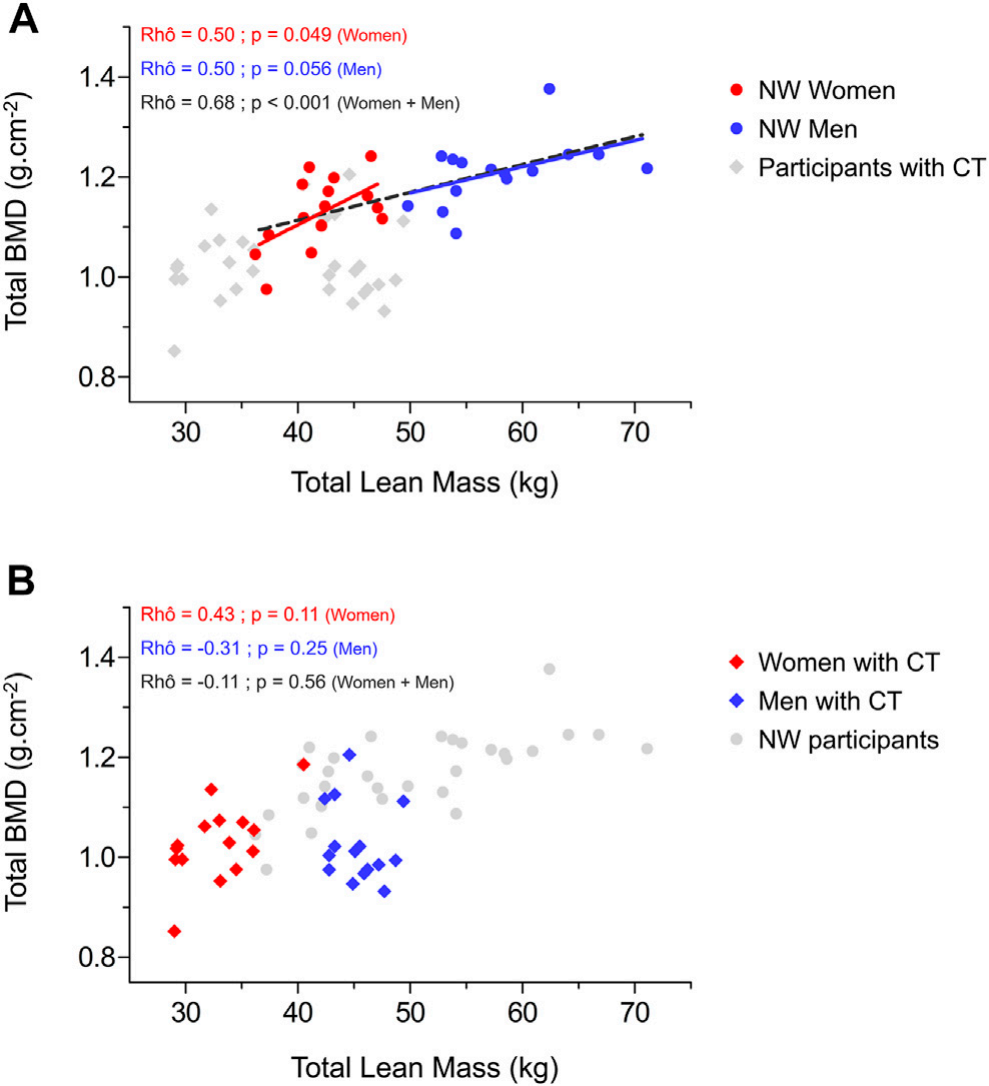
Relationship between total bone mineral density and total lean mass in normal weight women and men **(A)** and in women and men with constitutional thinness **(B)**. BMD, bone mineral density; CT, constitutional thinness; NW, normal weight.

Relationships between total and legs lean mass appeared strongly positively associated to most of the BMD variables in NW participants while almost no significant correlation was obtained for CT participants (Figure 2). This absence of correlation between lean mass and BMD from different region sites would be rather due to men with CT than to women with CT. Indeed, several negative moderate correlations were observed on those parameters for men with CT whereas weak to moderate positive associations were found for women with CT. As displayed in Figure 3, NW participants presented a strong correlation between their total BMD and total lean mass (rhô = 0.68, *p* < 0.001) whereas no correlation was observed in CT individuals. When distinguishing between sexes, NW women showed an almost continuous linear regression line with that of NW men (Figure 3A). Both NW women and men presented a moderate association between their total BMD and their total lean mass (respectively rhô = 0.50, *p* = 0.049 and rhô = 0.50, *p* = 0.056) while neither CT women nor CT men showed any association between these two variables (Figure 3). In accordance with these results, the parameters related to muscle fiber sizes appeared positively associated to different parameters of bone quality in NW participants contrary to individuals with CT for whom weak negative associations were observed (Figure 2).

IMTG and glycogen storage respectively appeared negatively and positively correlated to some bone quality variables in NW people contrary to CT individuals. Muscle parameters of capillary supply were observed positively associated to BMD of different region sites in NW participants while these associations were not observed in CT. This absence of association might be due to men with CT who presented negative rho values between muscle capillary supply parameters and bone parameters from the distal tibia (Supplementary Data S3B). No significant associations were observed between β-HAD enzyme and bone parameters in NW people whereas CT participants, and more specifically CT men, presented strong negative associations between these variables. No huge differences between CT and NW participants were obtained in relationships between enzymes of the mitochondrial respiratory chain and parameters of bone quality (Figure 2; Supplementary Data S2, S3), except for some negative strong correlations between CIV enzyme activity and BMD from microarchitecture analysis which were observed in CT women but not in NW women (Supplementary Data S2).

## 4 Discussion

The present study aimed to further investigate architecture, geometry, and texture of bone tissue in both women and men presenting CT, particularly questioning the relationships between bone and muscle structural organization.

The present analysis revealed a lower bone quality in CT vs. NW participants based on the results of 32 parameters among the 48 assessed; therefore demonstrating the consistency of this result. All the densitometry indexes and Z-scores from different region sites were found lower in CT (Table 1). HSA, which uses the properties of DXA images to derive hip geometric parameters associated with bone strength (Hind et al., 2012), also raised some concerns. Indeed, both HAL and CSMI that respectively represent a predictor of fracture risk and an estimate of bone resistance (Leslie et al., 2009) were found lower in CT, as well as femoral CSA (Table 1). As a lower TBS was observed compared with NW participants, bone texture in CT seems to be characterized by a poorly connected lacunar microarchitecture representative of a low mechanical resistance (Hans et al., 2011; Winzenrieth et al., 2012). Results of bone densitometry, HSA, and TBS—all recognized as particularly relevant in clinical practices—are therefore indicators of a potentially increased risk of fracture in CT population. Our previously published meta-analysis also showed lower vitamin D levels in CT (Bailly et al., 2021), which might explain our present results. Regarding the inexorable bone loss throughout life, present results obtained in a young population might be predictable of an increased risk of osteoporosis with ageing. Beyond bone quality assessments, it might be of major interest to further assess the prevalence of fractures in futures studies. While these results appeared alarming, other observations would rather qualify these concerns. For instance, neck shaft angle, also known to partly predict the risk of hip fracture (Ripamonti et al., 2014), was found similar between CT and NW participants. In addition, despite reaching significance, Z-scores should be balanced in the light of clinical approaches. No mean Z-scores below −1.0 were measured in CT (Table 1) and almost none of the participants presented any individual value below −2.0, representing an absence of osteopenic or osteoporotic Z-scores (ISCD, 2019), contrary to a previous study in which up to 44% of the CT women presented Z-scores < −2.0 (Galusca et al., 2008, 2009).

HR-pQCT of the distal tibia revealed an altered microarchitecture of this bone that plays a major role in supporting body weight (Table 2). All the dimensional parameters and all but one densitometry indexes were found lower in CT compared with NW individuals. These results also gave evidence of a smaller number of trabeculae, higher trabecular separations, and higher network inhomogeneity in CT (Table 2), suggesting a more fragile bone architecture. HR-pQCT of the distal radius (Table 3) allows the differentiation between a weight-supporting bone (distal tibia) with this non-weight-supporting bone (distal radius), which is of specific interest given that weight differences represent the major difference between CT and NW people. More than 20 kg distinguished CT from NW participants in the present study (Table 1) which might involve a much lower gravity constraint in CT vs. NW people. Contrary to the tibia microarchitecture, only dimensional parameters of distal radius were significantly lower in CT. Densities and structural organization of trabeculae did not appear lower in CT vs. NW people despite three tendencies (Table 3). Interestingly, the bone microarchitecture of non-weight-supporting bones appeared to be less impacted than weight-supporting bones in CT. This might suggest that the low bone quality in CT could only result from a low mechanical load induced by their low body weight. Based on this hypothesis, CT would be characterized by a “physiological” osteopenia reflecting the Frost’s mechanostat concept (Frost, 1987) which may not necessarily lead to an increased risks of fracture. Indeed, regarding their low body weight, a lower bone mechanical resistance might be required in case of falls to prevent fractures. According to Frost’s mechanostat, a strong association between body weight and total BMD was expected and was indeed observed in NW people. Nevertheless, no association was obtained in CT (Figure 2B), which tends to invalidate our hypothesis of a normal “physiological” weight-induced low BMD.

In the same time, an important part of the mechanical load comes from muscle strength in addition to the gravity force related to weight-supporting. Substantial physical loads are caused by muscle contractions, which explains the close relationship between bone strength and muscle force or size (Schoenau, 2005). Fifty-two percent of BMC variation would be explained by lean mass and only 20% by FM (Makovey et al., 2005; Guimarães et al., 2018). Although findings seem to slightly differ between studies, lean mass would be one of the main predictors of total BMD (Tagliaferri et al., 2015; Nguyen et al., 2020). A biomechanical reason explains why the muscle-derived forces represent an important source of mechanical loading—the proximity of muscle attachments to the motion axes of the bones results in very small lever arms which implies the development of important strengths to overcome this mechanical disadvantage (Avin et al., 2015). Even a very minor muscular deficit can induce a large effect on bone resorption (Hamrick, 2010). Space flight, bed rest, and osteoporosis experimentations have brought many evidence of the close relationships between these two interconnected tissues (Lloyd et al., 2014) deriving from the same embryogenic precursors cells (Shin et al., 2000). While NW participants indeed presented a strong positive correlation between total lean mass and total BMD, no association was however found in CT. These results might suggest a possible defect in the mechanical coupling between muscle and bone tissues, which contradicts the “muscle-bone” mechanical unit in CT and reinforces the hypothesis that bone impairments do not result from a “physiological” adaptation. If the low BMD in CT was not due to a lack of mechanical stimulation (disuse-induced bone losses), then it might result from an intrinsic disturbance of bone cells of a systematically-induced shift of the system’s setpoint (Ferretti et al., 2003). Yet, this assumption remains only speculative as not backed up by bone cellular activity related analyses.

The Figure 2 also supports this possible defect in the muscle-bone mechanical coupling in CT as many strong correlations between different lean mass and bone variables were found in NW participants contrary to individuals with CT. The major benefit of heatmaps (Figure 2; Supplementary Data S2, S3) consists in the robustness of the observations which are not only carried out between two variables but between various relative variables. Furthermore, these results were observed using different approaches (body composition analyses through DXA evaluations, HR-pQCT, and histology on muscle biopsies) which strengthen our confidence in the results interpretation (Figure 2). Beyond the mechanical coupling, muscle strength is also recognized for its action on bone metabolism (Guimarães et al., 2018). In order to investigate potential interactions between muscle metabolism and bone quality, our histological data from muscle biopsies (muscle storage, capillarization, enzyme activities) were cross-referenced with parameters of bone quality. If relationships appeared quite heterogeneous and difficult to interpret, we however observed distinct results between CT and NW participants (Figure 2). For instance, NW people presented positive associations between muscle capillarization and bone strength which appeared consistent with the hypothesis of some authors who proposed that blood flows to limb at a proportional level between bone and muscle tissues, and that a higher blood flow to bone might lead to an increase in bone resistance (Kaji, 2014). On the contrary, individuals with CT did not show these kinds of associations, suggesting once more impairments of their muscle-bone unit.

As a second main result, we observed a strong sex distinction for bone assessments in CT. Bone impairments appeared more pronounced in men than in women with CT. For both HR-pQCT of the tibia and the radius, many parameters of bone quality appeared lower in men with CT while not reaching significance in women with CT (Tables 2, 3). Despite being less impacted, women with CT still presented some bone impairments. Estrogen and androgen hormones, known to strongly regulate bone remodeling (Almeida et al., 2017), might have helped better understand these results. For women with CT, we previously demonstrated in a systematic review (Bailly et al., 2021) that estrogens, testosterone, dehydroepiandrosterone sulfate (DHEAS), follicle stimulating hormone (FSH), luteinizing hormone (LH), and sex hormone binding globulin (SHBG) were almost systematically found at normal levels (Germain et al., 2007, 2009, 2016; Galusca et al., 2008, 2012, 2015, 2018; Hasegawa et al., 2011; Estour et al., 2017). However, our meta-analysis also revealed in the same time that CT women presented significantly lower testosterone levels and tended to present lower DHEAS levels (Bailly et al., 2021). This moderately impaired sex hormone profile in CT women may partly explain their moderately altered bone quality. To the best of our knowledge, sexual hormones have however not been specifically assessed in CT men. Yet, we might formulate the hypothesis that if testosterone was already lower for CT women, men with CT may present an even lower testosterone level which could account for an important factor explaining their more pronounced bone impairments (Mohamad et al., 2016). Muscle-to-bone associations also highly differ between women and men with CT. Women with CT presented similar positive associations between muscle and bone parameters compared with NW women suggesting a normal mechanical coupling (Supplementary Data S2). Distinctly, men with CT did not present such correlations and even presented several negative significant correlations between muscle and bone parameters (Supplementary Data S3B). Altogether, present findings tend to point out that the mechanical coupling between women and men with CT is regulated in a different way. Although most of studies focused on women with CT, it appears essential to further study CT in men populations who may be at greater risk.

Some limitations can be identified in the present study. First, this study included a modest number of participants, and while many positive findings are evidenced, the absence of effect in some results may be related to this limited number of subjects. In addition, participants with CT would be slightly less physically active and older (+2.4 years) than NW people (Table 1) which might induce an effect on muscle-bone analyses. However, values do not seem highly different from a clinical point of view and we included these two parameters as a covariate to minimize a potential effect. Other important limitations are due to the fact that muscle-bone analyses were only a secondary outcome in the wide project from which this first muscle-bone analysis in CT originated. For instance, a direct assessment of muscle strength is missing in the present study. The interpretations are based on the implicit assumption that muscle mass or muscle fiber sizes which only provide anatomical and biochemical information are associated with muscle strength, and therefore with the mechanical constraint. However, we assume that this assumption remains inaccurate and that a direct muscle torque assessment in CT would have enabled a direct exploration beyond the structural aspects, either using this measure as a covariate or even as a specific outcome of interest. Lastly, only structural interactions were explored here whereas the endocrine crosstalk is also recognized as being fundamental in the muscle-bone functional unit. This reduced our ability to mechanistically discuss the present results from a biological point of view. Only three studies (Galusca et al., 2008, 2018; Estour et al., 2017) have assessed some of the main bone biomarkers to date. Although no major bone remodeling impairment was reported in these studies (Galusca et al., 2008, 2018; Estour et al., 2017), new protocols specifically designed to analyze bone issues should be performed, for instance further investigating bone hormones/bone remodeling markers (such as FGF23, osteocalcin, CTX, P1NP, OPG, RANKL, PTH) and mediators/myokines from muscle tissue (such as myostatin, irisin, osteoglycin, MMP-2, IGF-1, FGF-2, IL-6, IL-15). Indeed, our unexpected and uncommon observations call for further mechanistic explorations.

To conclude, present results showed that CT was characterized by a low bone quality in terms of densitometry, geometry, texture, and microarchitecture. These findings also suggested that weight-supporting bones might be more affected than non-weight-supporting bones, which therefore raised the hypothesis that the low bone quality might be induced by a low mechanical load. However, no correlation was found between total BMD with both body weight and lean mass in CT contrary to NW people. For the first time, these results highlighted a potential defect in the functional muscle-bone unit in CT. Another main finding was the high sex differences which have been observed. The bone deficits were more pronounced in CT men than in CT women. Results of correlations also tended to show that the mechanical muscle-bone coupling appeared relatively normal for women with CT contrary to men with CT who seemed to present highly impaired muscle-bone mechanical relationships. Regarding the importance of endocrine crosstalk between these two tissues, it might be of particular interest to focus on it in future studies in order to further mechanistically explain the present structural observations.

## Data Availability

The original contributions presented in the study are included in the article/Supplementary Material, further inquiries can be directed to the corresponding author.
