# Supplementary material for: New Insights on Bone Tissue and Structural Muscle-Bone Unit in Constitutional Thinness
Source: Front Physiol. 2022 Jul 8;13:921351. doi: 10.3389/fphys.2022.921351 (PMC9305386; doi:10.3389/fphys.2022.921351)
Supplement: Supplementary file 1 [file DataSheet1.docx]

Supplementary Material

# Supplementary Data

**Supplementary Data 1:** Conjugation of primary and secondary antibodies for fiber type and basal lamina identification.

| **Primary antibody** | | | | | | |  | **Secondary antibody** | | | | | |
| --- | --- | --- | --- | --- | --- | --- | --- | --- | --- | --- | --- | --- | --- |
| **Antibody** | **Reference** | **Species** | **Clonality** | **Isotype** | **Dilution** | **Source** |  | **Reference** | **Species** | **Isotype** | **Source** | **Dilution** |  |
| Anti-MHCI | BA-F8 –  AB_10572253 | Mouse | Mono | IgG2b | 1:100 | DSHB |  | 350 –  A-21140 | GaM | IgG2b | ThermoFisher  Alexa Fluor^®^ | 1:300 |  |
| Anti-MHC (all but IIX) | BF-35 –  AB_2274680 | Mouse | Mono | IgG1 | 1:100 | DSHB |  | 488 –  A-21121 | GaM | IgG1 | ThermoFisher  Alexa Fluor^®^ | 1:300 |  |
| Anti-laminin | 2E8 –  AB_2134060 | Mouse | Mono | IgG2a | 1:100 | DSHB |  | 633 –  A-21136 | GaM | IgG2a | ThermoFisher  Alexa Fluor^®^ | 1:300 |  |
| Anti-CD31 | Anti-CD31-JC70A | Mouse | Mono | IgG1 | 1:40 | Dako |  | 546 –  A-21123 | GaM | IgG1 | ThermoFisher  Alexa Fluor^®^ | 1:300 |  |

DSHB : developmental studies hybridoma bank, GaM : goat anti-mouse, MHC : myosin heavy chain


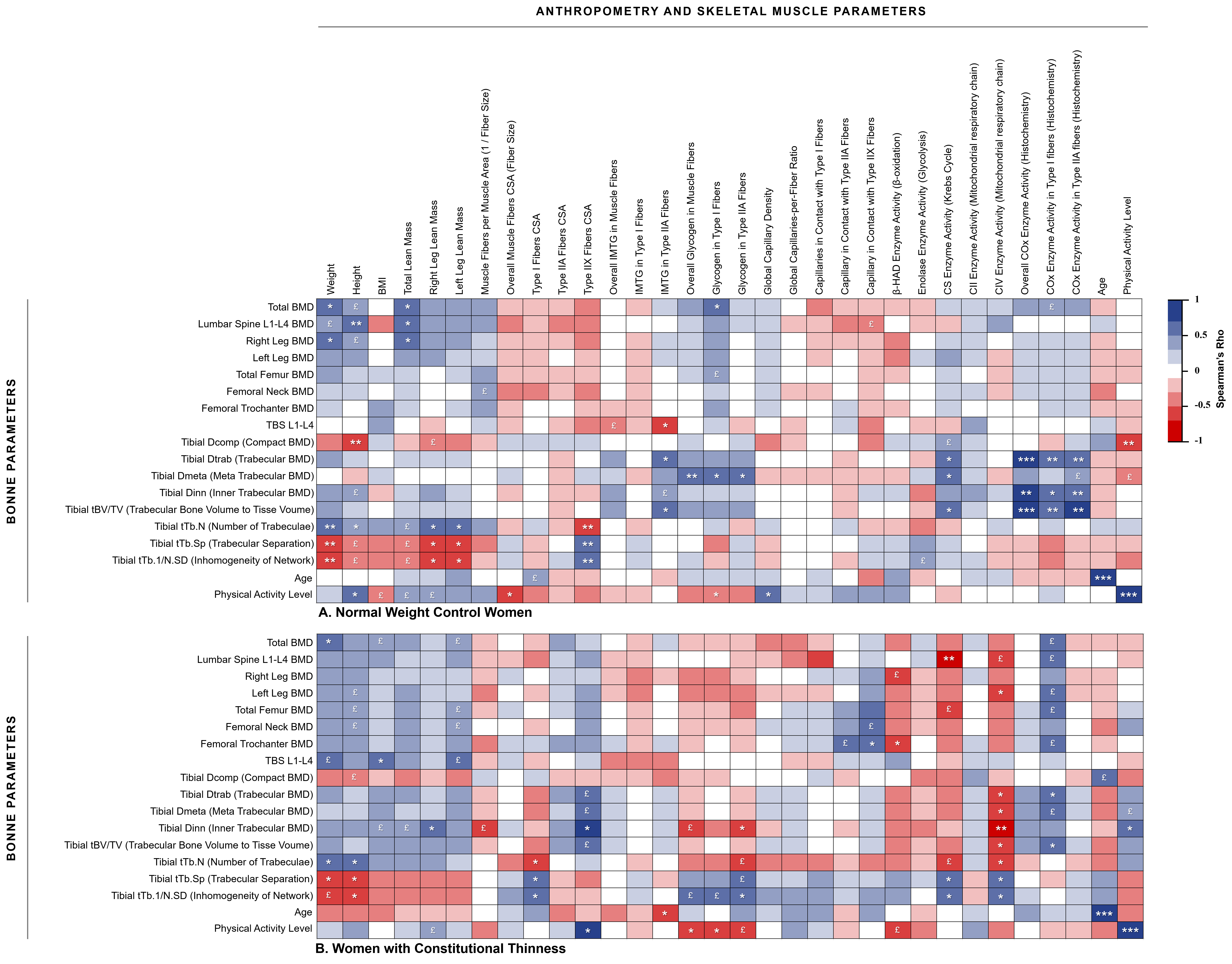


**Supplementary Data 2.** Heat map of correlations between muscle and bone assessments in women only. (**A**) Normal weight control women; (**B**) Women with constitutional thinness

* p<0.05, ** p<0.01, *** p<0.001, £ 0.05<p<0.1 (trend)

BMD: bone mineral density, BMI: body mass index, COx: cytochrome-c oxidase, CS: citrate synthase, CSA: cross-sectional area, IMTG: intramuscular triglycerides, TBS: trabecular bone score


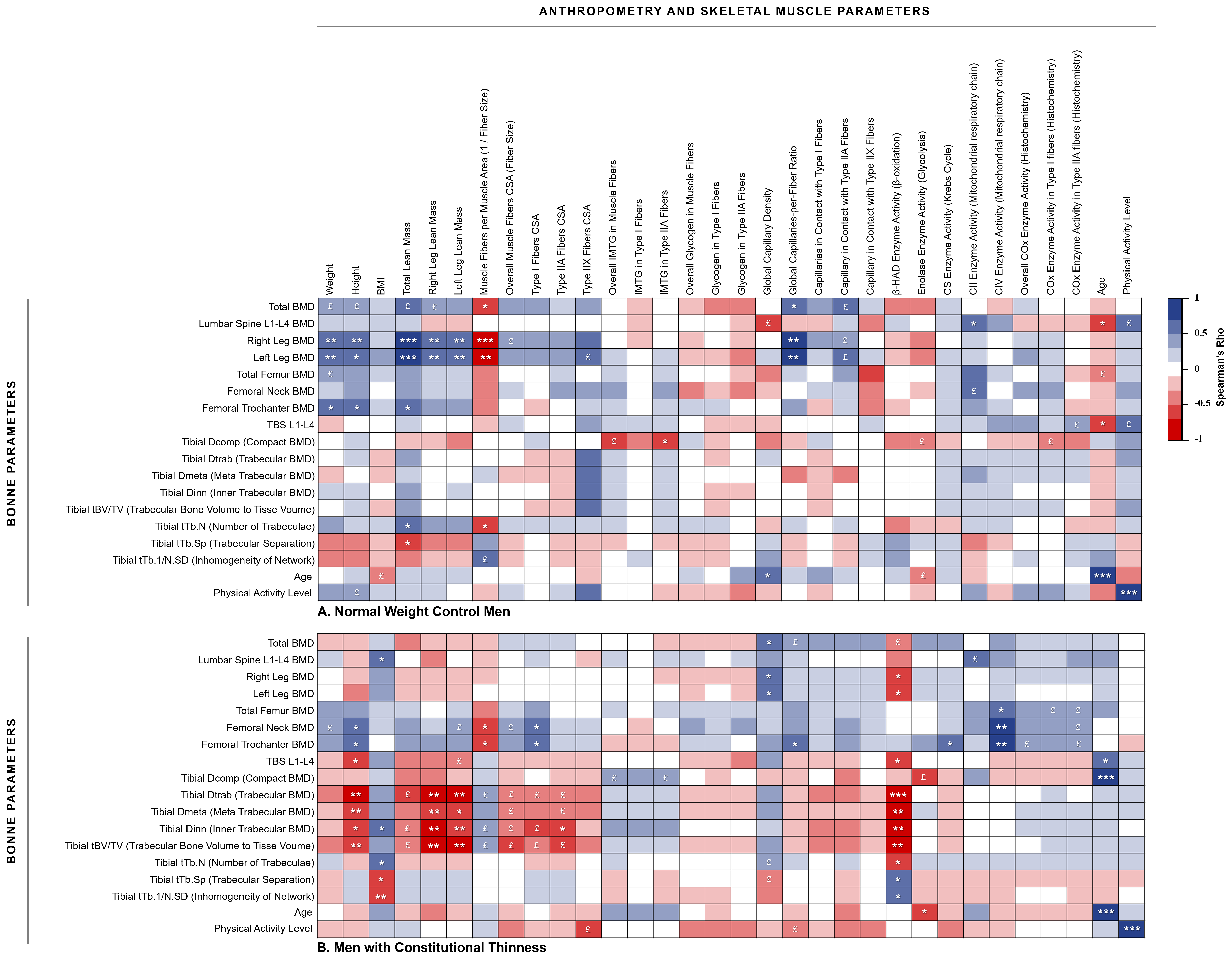


**Supplementary Data 3:** Heat map of correlations between muscle and bone assessments in men only. (**A**) Normal weight control men; (**B**) Men with constitutional thinness

* p<0.05, ** p<0.01, *** p<0.001, £ 0.05<p<0.1 (trend)

BMD: bone mineral density, BMI: body mass index, COx: cytochrome-c oxidase, CS: citrate synthase, CSA: cross-sectional area, IMTG: intramuscular triglycerides, TBS: trabecular bone score
